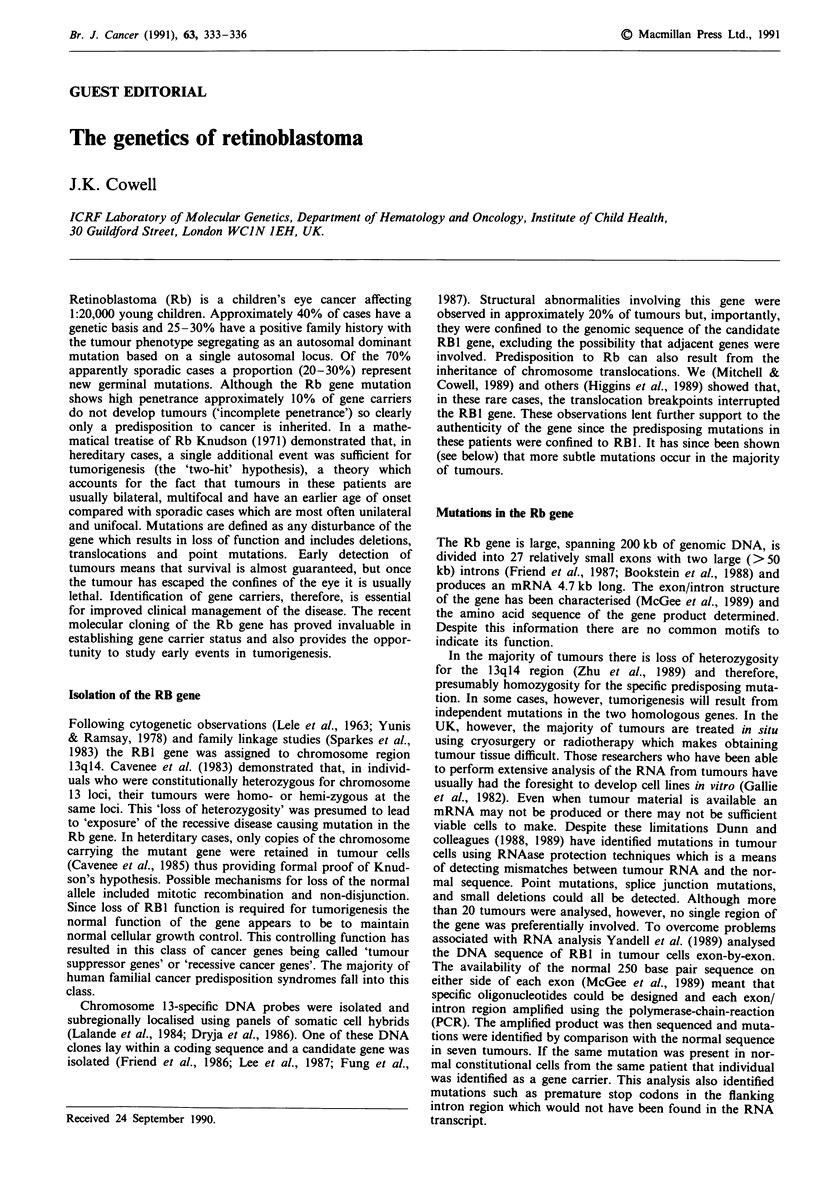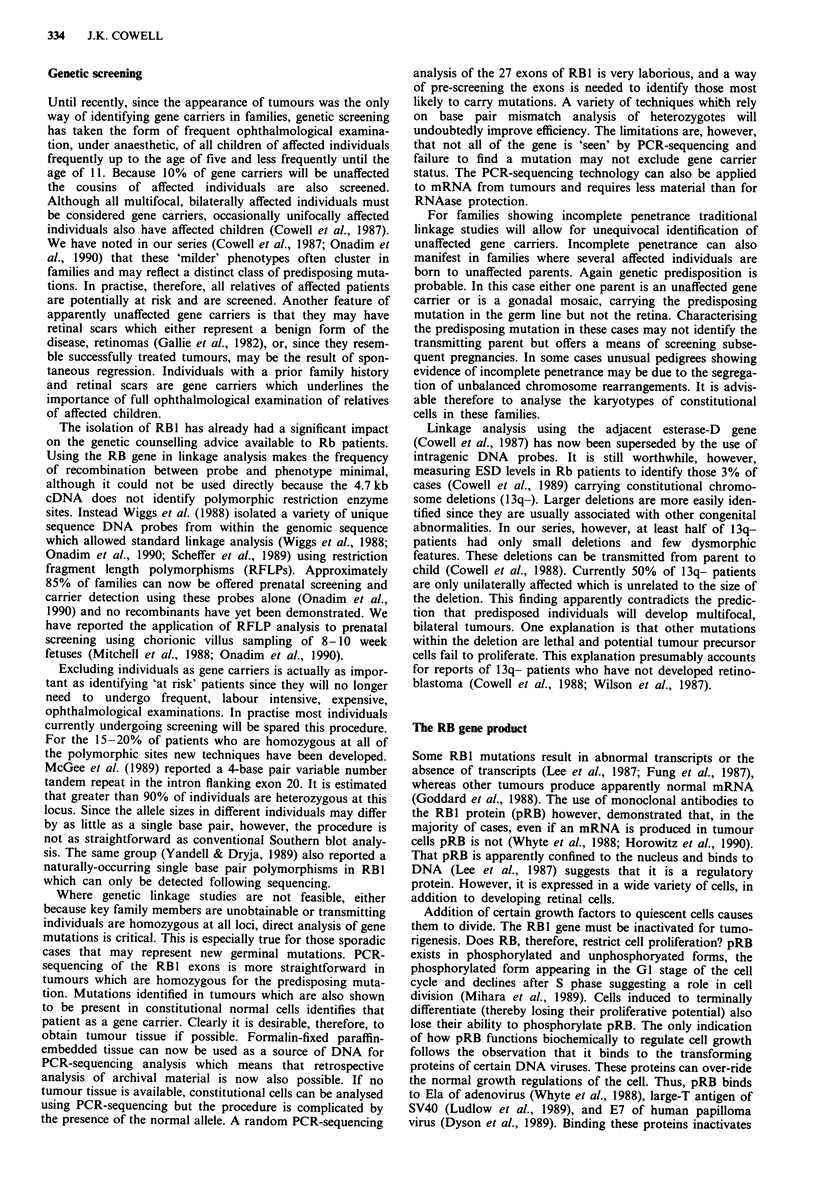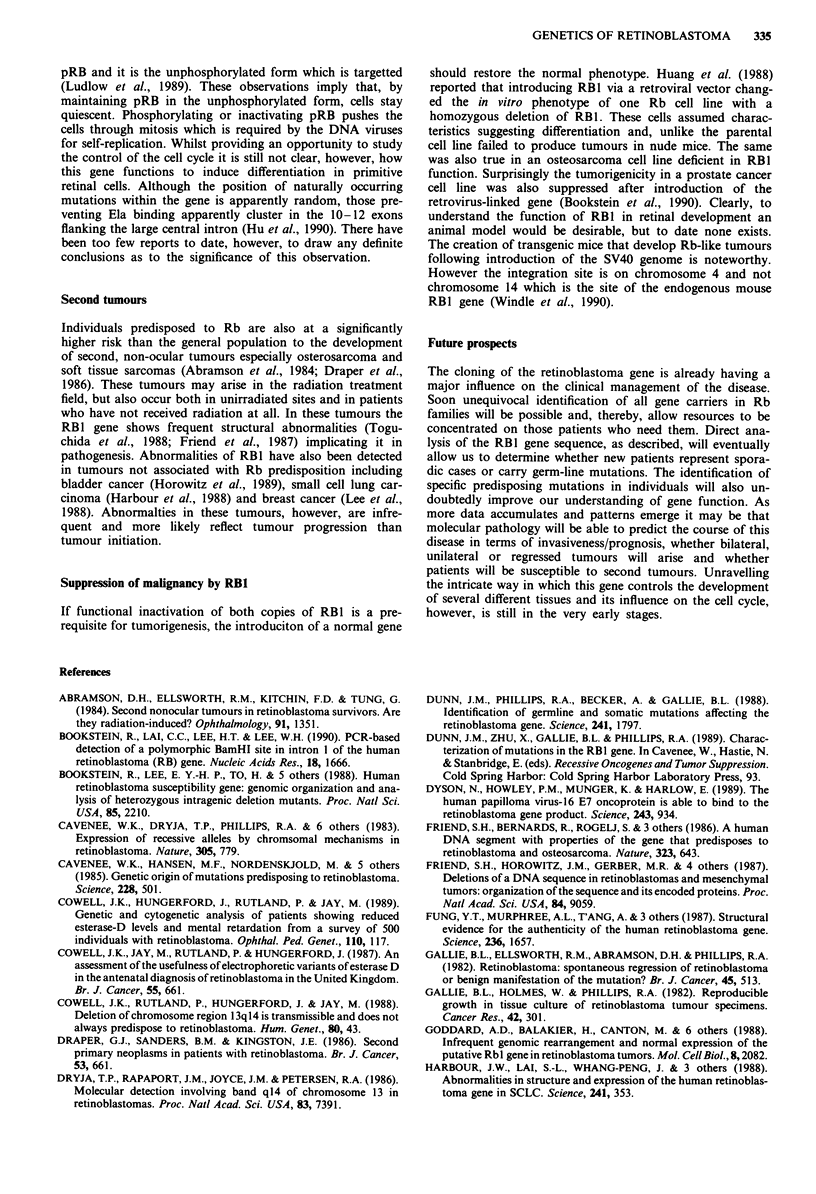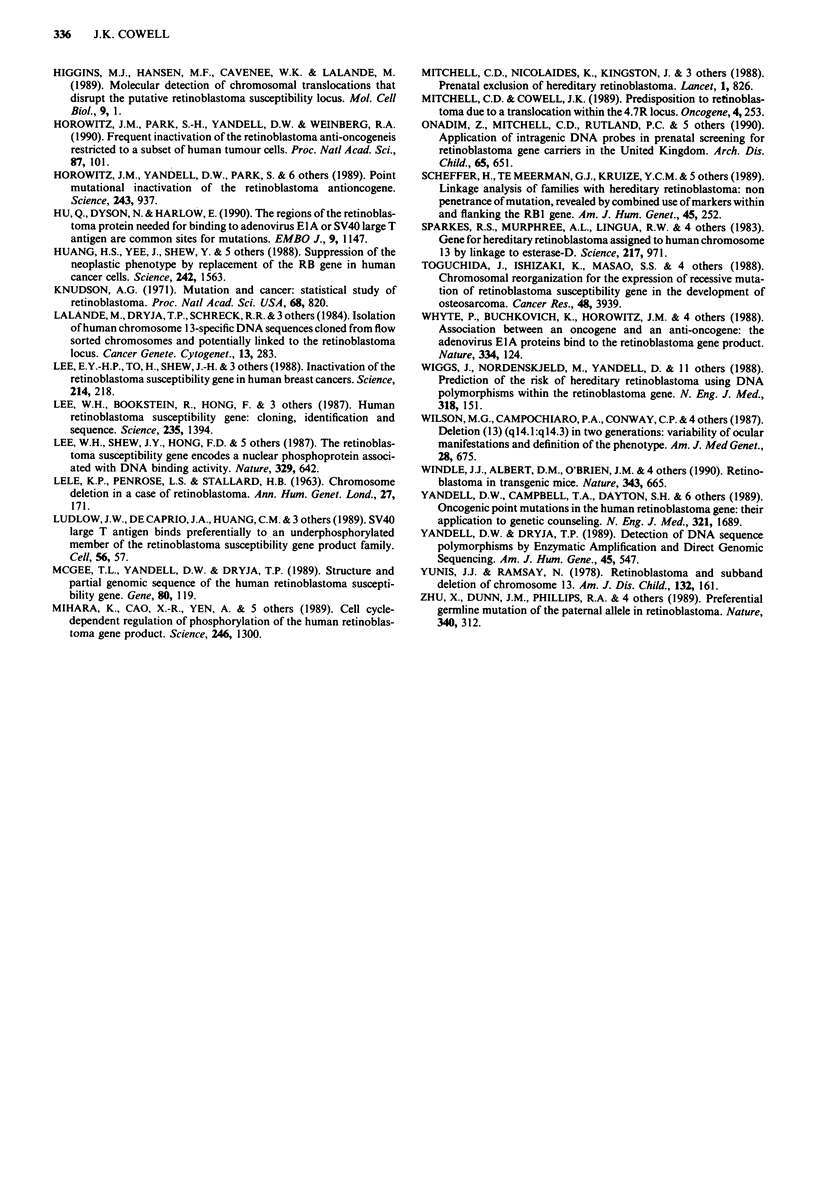# The genetics of retinoblastoma.

**DOI:** 10.1038/bjc.1991.79

**Published:** 1991-03

**Authors:** J. K. Cowell


Br. J. Cancer (1991), 63, 333–336 © Macmillan Press Ltd., 1991

GUEST EDITORIAL

### The genetics of retinoblastoma

J.K. Cowell

*ICRF Laboratory of Molecular Genetics, Department of Hematology and Oncology, Institute of Child Health, 30 Guildford Street, London WC1N 1EH, UK.*

Retinoblastoma (Rb) is a children's eye cancer affecting 1:20,000 young children. Approximately 40% of cases have a genetic basis and 25–30% have a positive family history with the tumour phenotype segregating as an autosomal dominant mutation based on a single autosomal locus. Of the 70% apparently sporadic cases a proportion (20–30%) represent new germinal mutations. Although the Rb gene mutation shows high penetrance approximately 10% of gene carriers do not develop tumours ('incomplete penetrance') so clearly only a predisposition to cancer is inherited. In a mathematical treatise of Rb Knudson (1971) demonstrated that, in hereditary cases, a single additional event was sufficient for tumorigenesis (the 'two-hit' hypothesis), a theory which accounts for the fact that tumours in these patients are usually bilateral, multifocal and have an earlier age of onset compared with sporadic cases which are most often unilateral and unifocal. Mutations are defined as any disturbance of the gene which results in loss of function and includes deletions, translocations and point mutations. Early detection of tumours means that survival is almost guaranteed, but once the tumour has escaped the confines of the eye it is usually lethal. Identification of gene carriers, therefore, is essential for improved clinical management of the disease. The recent molecular cloning of the Rb gene has proved invaluable in establishing gene carrier status and also provides the opportunity to study early events in tumorigenesis.

### Isolation of the RB gene

Following cytogenetic observations (Lele *et al.*, 1963; Yunis & Ramsay, 1978) and family linkage studies (Sparkes *et al.*, 1983) the RB1 gene was assigned to chromosome region 13q14. Cavenee *et al.* (1983) demonstrated that, in individuals who were constitutionally heterozygous for chromosome 13 loci, their tumours were homo- or hemi-zygous at the same loci. This 'loss of heterozygosity' was presumed to lead to 'exposure' of the recessive disease causing mutation in the Rb gene. In herditary cases, only copies of the chromosome carrying the mutant gene were retained in tumour cells (Cavenee *et al.*, 1985) thus providing formal proof of Knudson's hypothesis. Possible mechanisms for loss of the normal allele included mitotic recombination and non-disjunction. Since loss of RB1 function is required for tumorigenesis the normal function of the gene appears to be to maintain normal cellular growth control. This controlling function has resulted in this class of cancer genes being called 'tumour suppressor genes' or 'recessive cancer genes'. The majority of human familial cancer predisposition syndromes fall into this class.

Chromosome 13-specific DNA probes were isolated and subregionally localised using panels of somatic cell hybrids (Lalande *et al.*, 1984; Dryja *et al.*, 1986). One of these DNA clones lay within a coding sequence and a candidate gene was isolated (Friend *et al.*, 1986; Lee *et al.*, 1987; Fung *et al.*, 1987). Structural abnormalities involving this gene were observed in approximately 20% of tumours but, importantly, they were confined to the genomic sequence of the candidate RB1 gene, excluding the possibility that adjacent genes were involved. Predisposition to Rb can also result from the inheritance of chromosome translocations. We (Mitchell & Cowell, 1989) and others (Higgins *et al.*, 1989) showed that, in these rare cases, the translocation breakpoints interrupted the RB1 gene. These observations lent further support to the authenticity of the gene since the predisposing mutations in these patients were confined to RB1. It has since been shown (see below) that more subtle mutations occur in the majority of tumours.

### Mutations in the Rb gene

The Rb gene is large, spanning 200 kb of genomic DNA, is divided into 27 relatively small exons with two large (>50 kb) introns (Friend *et al.*, 1987; Bookstein *et al.*, 1988) and produces an mRNA 4.7 kb long. The exon/intron structure of the gene has been characterised (McGee *et al.*, 1989) and the amino acid sequence of the gene product determined. Despite this information there are no common motifs to indicate its function.

In the majority of tumours there is loss of heterozygosity for the 13q14 region (Zhu *et al.*, 1989) and therefore, presumably homozygosity for the specific predisposing mutation. In some cases, however, tumorigenesis will result from independent mutations in the two homologous genes. In the UK, however, the majority of tumours are treated *in situ* using cryosurgery or radiotherapy which makes obtaining tumour tissue difficult. Those researchers who have been able to perform extensive analysis of the RNA from tumours have usually had the foresight to develop cell lines *in vitro* (Gallie *et al.*, 1982). Even when tumour material is available an mRNA may not be produced or there may not be sufficient viable cells to make. Despite these limitations Dunn and colleagues (1988, 1989) have identified mutations in tumour cells using RNAase protection techniques which is a means of detecting mismatches between tumour RNA and the normal sequence. Point mutations, splice junction mutations, and small deletions could all be detected. Although more than 20 tumours were analysed, however, no single region of the gene was preferentially involved. To overcome problems associated with RNA analysis Yandell *et al.* (1989) analysed the DNA sequence of RB1 in tumour cells exon-by-exon. The availability of the normal 250 base pair sequence on either side of each exon (McGee *et al.*, 1989) meant that specific oligonucleotides could be designed and each exon/intron region amplified using the polymerase-chain-reaction (PCR). The amplified product was then sequenced and mutations were identified by comparison with the normal sequence in seven tumours. If the same mutation was present in normal constitutional cells from the same patient that individual was identified as a gene carrier. This analysis also identified mutations such as premature stop codons in the flanking intron region which would not have been found in the RNA transcript.

Received 24 September 1990.

### Genetic screening

Until recently, since the appearance of tumours was the only way of identifying gene carriers in families, genetic screening has taken the form of frequent ophthalmological examination, under anaesthetic, of all children of affected individuals frequently up to the age of five and less frequently until the age of 11. Because 10% of gene carriers will be unaffected the cousins of affected individuals are also screened. Although all multifocal, bilaterally affected individuals must be considered gene carriers, occasionally unifocally affected individuals also have affected children (Cowell *et al.*, 1987). We have noted in our series (Cowell *et al.*, 1987; Onadim *et al.*, 1990) that these 'milder' phenotypes often cluster in families and may reflect a distinct class of predisposing mutations. In practise, therefore, all relatives of affected patients are potentially at risk and are screened. Another feature of apparently unaffected gene carriers is that they may have retinal scars which either represent a benign form of the disease, retinomas (Gallie *et al.*, 1982), or, since they resemble successfully treated tumours, may be the result of spontaneous regression. Individuals with a prior family history and retinal scars are gene carriers which underlines the importance of full ophthalmological examination of relatives of affected children.

The isolation of RB1 has already had a significant impact on the genetic counselling advice available to Rb patients. Using the RB gene in linkage analysis makes the frequency of recombination between probe and phenotype minimal, although it could not be used directly because the 4.7 kb cDNA does not identify polymorphic restriction enzyme sites. Instead Wiggs *et al.* (1988) isolated a variety of unique sequence DNA probes from within the genomic sequence which allowed standard linkage analysis (Wiggs *et al.*, 1988; Onadim *et al.*, 1990; Scheffer *et al.*, 1989) using restriction fragment length polymorphisms (RFLPs). Approximately 85% of families can now be offered prenatal screening and carrier detection using these probes alone (Onadim *et al.*, 1990) and no recombinants have yet been demonstrated. We have reported the application of RFLP analysis to prenatal screening using chorionic villus sampling of 8–10 week fetuses (Mitchell *et al.*, 1988; Onadim *et al.*, 1990).

Excluding individuals as gene carriers is actually as important as identifying 'at risk' patients since they will no longer need to undergo frequent, labour intensive, expensive, ophthalmological examinations. In practise most individuals currently undergoing screening will be spared this procedure. For the 15–20% of patients who are homozygous at all of the polymorphic sites new techniques have been developed. McGee *et al.* (1989) reported a 4-base pair variable number tandem repeat in the intron flanking exon 20. It is estimated that greater than 90% of individuals are heterozygous at this locus. Since the allele sizes in different individuals may differ by as little as a single base pair, however, the procedure is not as straightforward as conventional Southern blot analysis. The same group (Yandell & Dryja, 1989) also reported a naturally-occurring single base pair polymorphisms in RB1 which can only be detected following sequencing.

Where genetic linkage studies are not feasible, either because key family members are unobtainable or transmitting individuals are homozygous at all loci, direct analysis of gene mutations is critical. This is especially true for those sporadic cases that may represent new germinal mutations. PCR-sequencing of the RB1 exons is more straightforward in tumours which are homozygous for the predisposing mutation. Mutations identified in tumours which are also shown to be present in constitutional normal cells identifies that patient as a gene carrier. Clearly it is desirable, therefore, to obtain tumour tissue if possible. Formalin-fixed paraffin-embedded tissue can now be used as a source of DNA for PCR-sequencing analysis which means that retrospective analysis of archival material is now also possible. If no tumour tissue is available, constitutional cells can be analysed using PCR-sequencing but the procedure is complicated by the presence of the normal allele. A random PCR-sequencing analysis of the 27 exons of RB1 is very laborious, and a way of pre-screening the exons is needed to identify those most likely to carry mutations. A variety of techniques which rely on base pair mismatch analysis of heterozygotes will undoubtedly improve efficiency. The limitations are, however, that not all of the gene is 'seen' by PCR-sequencing and failure to find a mutation may not exclude gene carrier status. The PCR-sequencing technology can also be applied to mRNA from tumours and requires less material than for RNAase protection.

For families showing incomplete penetrance traditional linkage studies will allow for unequivocal identification of unaffected gene carriers. Incomplete penetrance can also manifest in families where several affected individuals are born to unaffected parents. Again genetic predisposition is probable. In this case either one parent is an unaffected gene carrier or is a gonadal mosaic, carrying the predisposing mutation in the germ line but not the retina. Characterising the predisposing mutation in these cases may not identify the transmitting parent but offers a means of screening subsequent pregnancies. In some cases unusual pedigrees showing evidence of incomplete penetrance may be due to the segregation of unbalanced chromosome rearrangements. It is advisable therefore to analyse the karyotypes of constitutional cells in these families.

Linkage analysis using the adjacent esterase-D gene (Cowell *et al.*, 1987) has now been superseded by the use of intragenic DNA probes. It is still worthwhile, however, measuring ESD levels in Rb patients to identify those 3% of cases (Cowell *et al.*, 1989) carrying constitutional chromosome deletions (13q–). Larger deletions are more easily identified since they are usually associated with other congenital abnormalities. In our series, however, at least half of 13q– patients had only small deletions and few dysmorphic features. These deletions can be transmitted from parent to child (Cowell *et al.*, 1988). Currently 50% of 13q– patients are only unilaterally affected which is unrelated to the size of the deletion. This finding apparently contradicts the prediction that predisposed individuals will develop multifocal, bilateral tumours. One explanation is that other mutations within the deletion are lethal and potential tumour precursor cells fail to proliferate. This explanation presumably accounts for reports of 13q– patients who have not developed retinoblastoma (Cowell *et al.*, 1988; Wilson *et al.*, 1987).

### The RB gene product

Some RB1 mutations result in abnormal transcripts or the absence of transcripts (Lee *et al.*, 1987; Fung *et al.*, 1987), whereas other tumours produce apparently normal mRNA (Goddard *et al.*, 1988). The use of monoclonal antibodies to the RB1 protein (pRB) however, demonstrated that, in the majority of cases, even if an mRNA is produced in tumour cells pRB is not (Whyte *et al.*, 1988; Horowitz *et al.*, 1990). That pRB is apparently confined to the nucleus and binds to DNA (Lee *et al.*, 1987) suggests that it is a regulatory protein. However, it is expressed in a wide variety of cells, in addition to developing retinal cells.

Addition of certain growth factors to quiescent cells causes them to divide. The RB1 gene must be inactivated for tumorigenesis. Does RB, therefore, restrict cell proliferation? pRB exists in phosphorylated and unphosphoryated forms, the phosphorylated form appearing in the G1 stage of the cell cycle and declines after S phase suggesting a role in cell division (Mihara *et al.*, 1989). Cells induced to terminally differentiate (thereby losing their proliferative potential) also lose their ability to phosphorylate pRB. The only indication of how pRB functions biochemically to regulate cell growth follows the observation that it binds to the transforming proteins of certain DNA viruses. These proteins can over-ride the normal growth regulations of the cell. Thus, pRB binds to Ela of adenovirus (Whyte *et al.*, 1988), large-T antigen of SV40 (Ludlow *et al.*, 1989), and E7 of human papilloma virus (Dyson *et al.*, 1989). Binding these proteins inactivates pRB and it is the unphosphorylated form which is targetted (Ludlow *et al.*, 1989). These observations imply that, by maintaining pRB in the unphosphorylated form, cells stay quiescent. Phosphorylating or inactivating pRB pushes the cells through mitosis which is required by the DNA viruses for self-replication. Whilst providing an opportunity to study the control of the cell cycle it is still not clear, however, how this gene functions to induce differentiation in primitive retinal cells. Although the position of naturally occurring mutations within the gene is apparently random, those preventing Ela binding apparently cluster in the 10–12 exons flanking the large central intron (Hu *et al.*, 1990). There have been too few reports to date, however, to draw any definite conclusions as to the significance of this observation.

### Second tumours

Individuals predisposed to Rb are also at a significantly higher risk than the general population to the development of second, non-ocular tumours especially osterosarcoma and soft tissue sarcomas (Abramson *et al.*, 1984; Draper *et al.*, 1986). These tumours may arise in the radiation treatment field, but also occur both in unirradiated sites and in patients who have not received radiation at all. In these tumours the RB1 gene shows frequent structural abnormalities (Toguchida *et al.*, 1988; Friend *et al.*, 1987) implicating it in pathogenesis. Abnormalities of RB1 have also been detected in tumours not associated with Rb predisposition including bladder cancer (Horowitz *et al.*, 1989), small cell lung carcinoma (Harbour *et al.*, 1988) and breast cancer (Lee *et al.*, 1988). Abnormalties in these tumours, however, are infrequent and more likely reflect tumour progression than tumour initiation.

### Suppression of malignancy by RB1

If functional inactivation of both copies of RB1 is a prerequisite for tumorigenesis, the introduciton of a normal gene should restore the normal phenotype. Huang *et al.* (1988) reported that introducing RB1 via a retroviral vector changed the *in vitro* phenotype of one Rb cell line with a homozygous deletion of RB1. These cells assumed characteristics suggesting differentiation and, unlike the parental cell line failed to produce tumours in nude mice. The same was also true in an osteosarcoma cell line deficient in RB1 function. Surprisingly the tumorigenicity in a prostate cancer cell line was also suppressed after introduction of the retrovirus-linked gene (Bookstein *et al.*, 1990). Clearly, to understand the function of RB1 in retinal development an animal model would be desirable, but to date none exists. The creation of transgenic mice that develop Rb-like tumours following introduction of the SV40 genome is noteworthy. However the integration site is on chromosome 4 and not chromosome 14 which is the site of the endogenous mouse RB1 gene (Windle *et al.*, 1990).

### Future prospects

The cloning of the retinoblastoma gene is already having a major influence on the clinical management of the disease. Soon unequivocal identification of all gene carriers in Rb families will be possible and, thereby, allow resources to be concentrated on those patients who need them. Direct analysis of the RB1 gene sequence, as described, will eventually allow us to determine whether new patients represent sporadic cases or carry germ-line mutations. The identification of specific predisposing mutations in individuals will also undoubtedly improve our understanding of gene function. As more data accumulates and patterns emerge it may be that molecular pathology will be able to predict the course of this disease in terms of invasiveness/prognosis, whether bilateral, unilateral or regressed tumours will arise and whether patients will be susceptible to second tumours. Unravelling the intricate way in which this gene controls the development of several different tissues and its influence on the cell cycle, however, is still in the very early stages.

### References

ABRAMSON, D.H., ELLSWORTH, R.M., KITCHIN, F.D. & TUNG, G. (1984). Second nonocular tumours in retinoblastoma survivors. Are they radiation-induced? *Ophthalmology*, **91**, 1351.

BOOKSTEIN, R., LAI, C.C., LEE, H.T. & LEE, W.H. (1990). PCR-based detection of a polymorphic BamHI site in intron 1 of the human retinoblastoma (RB) gene. *Nucleic Acids Res.*, **18**, 1666.

BOOKSTEIN, R., LEE, E. Y.-H. P., TO, H. & 5 others (1988). Human retinoblastoma susceptibility gene: genomic organization and analysis of heterozygous intragenic deletion mutants. *Proc. Natl Sci. USA*, **85**, 2210.

CAVENEE, W.K., DRYJA, T.P., PHILLIPS, R.A. & 6 others (1983). Expression of recessive alleles by chromsomal mechanisms in retinoblastoma. *Nature*, **305**, 779.

CAVENEE, W.K., HANSEN, M.F., NORDENSKJOLD, M. & 5 others (1985). Genetic origin of mutations predisposing to retinoblastoma. *Science*, **228**, 501.

COWELL, J.K., HUNGERFORD, J., RUTLAND, P. & JAY, M. (1989). Genetic and cytogenetic analysis of patients showing reduced esterase-D levels and mental retardation from a survey of 500 individuals with retinoblastoma. *Ophthal. Ped. Genet.*, **110**, 117.

COWELL, J.K., JAY, M., RUTLAND, P. & HUNGERFORD, J. (1987). An assessment of the usefulness of electrophoretic variants of esterase D in the antenatal diagnosis of retinoblastoma in the United Kingdom. *Br. J. Cancer*, **55**, 661.

COWELL, J.K., RUTLAND, P., HUNGERFORD, J. & JAY, M. (1988). Deletion of chromosome region 13q14 is transmissible and does not always predispose to retinoblastoma. *Hum. Genet.*, **80**, 43.

DRAPER, G.J., SANDERS, B.M. & KINGSTON, J.E. (1986). Second primary neoplasms in patients with retinoblastoma. *Br. J. Cancer*, **53**, 661.

DRYJA, T.P., RAPAPORT, J.M., JOYCE, J.M. & PETERSEN, R.A. (1986). Molecular detection involving band q14 of chromosome 13 in retinoblastomas. *Proc. Natl Acad. Sci. USA*, **83**, 7391.

DUNN, J.M., PHILLIPS, R.A., BECKER, A. & GALLIE, B.L. (1988). Identification of germline and somatic mutations affecting the retinoblastoma gene. *Science*, **241**, 1797.

DUNN, J.M., ZHU, X., GALLIE, B.L. & PHILLIPS, R.A. (1989). Characterization of mutations in the RB1 gene. In Cavenee, W., Hastie, N. & Stanbridge, E. (eds). *Recessive Oncogenes and Tumor Suppression*. Cold Spring Harbor: Cold Spring Harbor Laboratory Press, 93.

DYSON, N., HOWLEY, P.M., MUNGER, K. & HARLOW, E. (1989). The human papilloma virus-16 E7 oncoprotein is able to bind to the retinoblastoma gene product. *Science*, **243**, 934.

FRIEND, S.H., BERNARDS, R., ROGELJ, S. & 3 others (1986). A human DNA segment with properties of the gene that predisposes to retinoblastoma and osteosarcoma. *Nature*, **323**, 643.

FRIEND, S.H., HOROWITZ, J.M., GERBER, M.R. & 4 others (1987). Deletions of a DNA sequence in retinoblastomas and mesenchymal tumors: organization of the sequence and its encoded proteins. *Proc. Natl Acad. Sci. USA*, **84**, 9059.

FUNG, Y.T., MURPHREE, A.L., T'ANG, A. & 3 others (1987). Structural evidence for the authenticity of the human retinoblastoma gene. *Science*, **236**, 1657.

GALLIE, B.L., ELLSWORTH, R.M., ABRAMSON, D.H. & PHILLIPS, R.A. (1982). Retinoblastoma: spontaneous regression of retinoblastoma or benign manifestation of the mutation? *Br. J. Cancer*, **45**, 513.

GALLIE, B.L., HOLMES, W. & PHILLIPS, R.A. (1982). Reproducible growth in tissue culture of retinoblastoma tumour specimens. *Cancer Res.*, **42**, 301.

GODDARD, A.D., BALAKIER, H., CANTON, M. & 6 others (1988). Infrequent genomic rearrangement and normal expression of the putative Rb1 gene in retinoblastoma tumors. *Mol. Cell Biol.*, **8**, 2082.

HARBOUR, J.W., LAI, S.-L., WHANG-PENG, J. & 3 others (1988). Abnormalities in structure and expression of the human retinoblastoma gene in SCLC. *Science*, **241**, 353.

HIGGINS, M.J., HANSEN, M.F., CAVENEE, W.K. & LALANDE, M. (1989). Molecular detection of chromosomal translocations that disrupt the putative retinoblastoma susceptibility locus. *Mol. Cell Biol.*, **9**, 1.

HOROWITZ, J.M., PARK, S.-H., YANDELL, D.W. & WEINBERG, R.A. (1990). Frequent inactivation of the retinoblastoma anti-oncogeneis restricted to a subset of human tumour cells. *Proc. Natl Acad. Sci.*, **87**, 101.

HOROWITZ, J.M., YANDELL, D.W., PARK, S. & 6 others (1989). Point mutational inactivation of the retinoblastoma antioncogene. *Science*, **243**, 937.

HU, Q., DYSON, N. & HARLOW, E. (1990). The regions of the retinoblastoma protein needed for binding to adenovirus E1A or SV40 large T antigen are common sites for mutations. *EMBO J.*, **9**, 1147.

HUANG, H.S., YEE, J., SHEW, Y. & 5 others (1988). Suppression of the neoplastic phenotype by replacement of the RB gene in human cancer cells. *Science*, **242**, 1563.

KNUDSON, A.G. (1971). Mutation and cancer: statistical study of retinoblastoma. *Proc. Natl Acad. Sci. USA*, **68**, 820.

LALANDE, M., DRYJA, T.P., SCHRECK, R.R. & 3 others (1984). Isolation of human chromosome 13-specific DNA sequences cloned from flow sorted chromosomes and potentially linked to the retinoblastoma locus. *Cancer Genete. Cytogenet.*, **13**, 283.

LEE, E.Y.-H.P., TO, H., SHEW, J.-H. & 3 others (1988). Inactivation of the retinoblastoma susceptibility gene in human breast cancers. *Science*, **214**, 218.

LEE, W.H., BOOKSTEIN, R., HONG, F. & 3 others (1987). Human retinoblastoma susceptibility gene: cloning, identification and sequence. *Science*, **235**, 1394.

LEE, W.H., SHEW, J.Y., HONG, F.D. & 5 others (1987). The retinoblastoma susceptibility gene encodes a nuclear phosphoprotein associated with DNA binding activity. *Nature*, **329**, 642.

LELE, K.P., PENROSE, L.S. & STALLARD, H.B. (1963). Chromosome deletion in a case of retinoblastoma. *Ann. Hum. Genet. Lond.*, **27**, 171.

LUDLOW, J.W., DE CAPRIO, J.A., HUANG, C.M. & 3 others (1989). SV40 large T antigen binds preferentially to an underphosphorylated member of the retinoblastoma susceptibility gene product family. *Cell*, **56**, 57.

MCGEE, T.L., YANDELL, D.W. & DRYJA, T.P. (1989). Structure and partial genomic sequence of the human retinoblastoma susceptibility gene. *Gene*, **80**, 119.

MIHARA, K., CAO, X.-R., YEN, A. & 5 others (1989). Cell cycle-dependent regulation of phosphorylation of the human retinoblastoma gene product. *Science*, **246**, 1300.

MITCHELL, C.D., NICOLAIDES, K., KINGSTON, J. & 3 others (1988). Prenatal exclusion of hereditary retinoblastoma. *Lancet*, **1**, 826.

MITCHELL, C.D. & COWELL, J.K. (1989). Predisposition to retinoblastoma due to a translocation within the 4.7R locus. *Oncogene*, **4**, 253.

ONADIM, Z., MITCHELL, C.D., RUTLAND, P.C. & 5 others (1990). Application of intragenic DNA probes in prenatal screening for retinoblastoma gene carriers in the United Kingdom. *Arch. Dis. Child.*, **65**, 651.

SCHEFFER, H., TE MEERMAN, G.J., KRUIZE, Y.C.M. & 5 others (1989). Linkage analysis of families with hereditary retinoblastoma: non penetrance of mutation, revealed by combined use of markers within and flanking the RB1 gene. *Am. J. Hum. Genet.*, **45**, 252.

SPARKES, R.S., MURPHREE, A.L., LINGUA, R.W. & 4 others (1983). Gene for hereditary retinoblastoma assigned to human chromosome 13 by linkage to esterase-D. *Science*, **217**, 971.

TOGUCHIDA, J., ISHIZAKI, K., MASAO, S.S. & 4 others (1988). Chromosomal reorganization for the expression of recessive mutation of retinoblastoma susceptibility gene in the development of osteosarcoma. *Cancer Res.*, **48**, 3939.

WHYTE, P., BUCHKOVICH, K., HOROWITZ, J.M. & 4 others (1988). Association between an oncogene and an anti-oncogene: the adenovirus E1A proteins bind to the retinoblastoma gene product. *Nature*, **334**, 124.

WIGGS, J., NORDENSKJELD, M., YANDELL, D. & 11 others (1988). Prediction of the risk of hereditary retinoblastoma using DNA polymorphisms within the retinoblastoma gene. *N. Eng. J. Med.*, **318**, 151.

WILSON, M.G., CAMPOCHIARO, P.A., CONWAY, C.P. & 4 others (1987). Deletion (13) (q14.1:q14.3) in two generations: variability of ocular manifestations and definition of the phenotype. *Am. J. Med Genet.*, **28**, 675.

WINDLE, J.J., ALBERT, D.M., O'BRIEN, J.M. & 4 others (1990). Retinoblastoma in transgenic mice. *Nature*, **343**, 665.

YANDELL, D.W., CAMPBELL, T.A., DAYTON, S.H. & 6 others (1989). Oncogenic point mutations in the human retinoblastoma gene: their application to genetic counseling. *N. Eng. J. Med.*, **321**, 1689.

YANDELL, D.W. & DRYJA, T.P. (1989). Detection of DNA sequence polymorphisms by Enzymatic Amplification and Direct Genomic Sequencing. *Am. J. Hum. Gene.*, **45**, 547.

YUNIS, J.J. & RAMSAY, N. (1978). Retinoblastoma and subband deletion of chromosome 13. *Am. J. Dis. Child.*, **132**, 161.

ZHU, X., DUNN, J.M., PHILLIPS, R.A. & 4 others (1989). Preferential germline mutation of the paternal allele in retinoblastoma. *Nature*, **340**, 312.